# Relationships between Long-Term Ozone Exposure and Allergic Rhinitis and Bronchitic Symptoms in Chinese Children

**DOI:** 10.3390/toxics9090221

**Published:** 2021-09-14

**Authors:** Pei-En Zhou, Zhengmin (Min) Qian, Stephen Edward McMillin, Michael G. Vaughn, Zhong-Yue Xie, Yu-Jie Xu, Li-Zi Lin, Li-Wen Hu, Bo-Yi Yang, Xiao-Wen Zeng, Wang-Jian Zhang, Ru-Qing Liu, Gongbo Chen, Guang-Hui Dong

**Affiliations:** 1 Guangdong Provincial Engineering Technology Research Center of Environmental Pollution and Health Risk Assessment, Department of Occupational and Environmental Health, School of Public Health, Sun Yat-sen University, Guangzhou 510080, China; zhoupen@mail2.sysu.edu.cn (P.-E.Z.); xiezhy35@mail2.sysu.edu.cn (Z.-Y.X.); xuyj59@mail2.sysu.edu.cn (Y.-J.X.); linlz@mail.sysu.edu.cn (L.-Z.L.); huliwen@mail.sysu.edu.cn (L.-W.H.); yangby23@mail.sysu.edu.cn (B.-Y.Y.); zxw63@mail.sysu.edu.cn (X.-W.Z.); zhangwj227@mail.sysu.edu.cn (W.-J.Z.); liurq@mail.sysu.edu.cn (R.-Q.L.); 2 Department of Epidemiology and Biostatistics, College for Public Health & Social Justice, Saint Louis University, Saint Louis, MO 63104, USA; zhengmin.qian@slu.edu; 3 School of Social Work, College for Public Health & Social Justice, Saint Louis University, Saint Louis, MO 63104, USA; stephen.mcmillin@slu.edu (S.E.M.); michael.vaughn@slu.edu (M.G.V.)

**Keywords:** allergic rhinitis, ozone, children and adolescent, China

## Abstract

Numerous studies have demonstrated that exposure to ambient ozone (O_3_) could have adverse effects on children’s respiratory health. However, previous studies mainly focused on asthma and wheezing. Evidence for allergic rhinitis and bronchitic symptoms (e.g., persistent cough and phlegm) associated with O_3_ is limited, and results from existing studies are inconsistent. This study included a total of 59,754 children from the seven northeastern cities study (SNEC), who were aged 2 to 17 years and from 94 kindergarten, elementary and middle schools. Information on doctor-diagnosed allergic rhinitis (AR), persistent cough, and persistent phlegm was collected during 2012–2013 using a standardized questionnaire developed by the American Thoracic Society (ATS). Information for potential confounders was also collected via questionnaire. Individuals’ exposure to ambient ozone (O_3_) during the four years before the investigation was estimated using a satellite-based random forest model. A higher level of O_3_ was significantly associated with increased risk of AR and bronchitic symptoms. After controlling for potential confounders, the OR (95% CI) were 1.13 (1.07–1.18), 1.10 (1.06–1.16), and 1.12 (1.05–1.20) for AR, persistent cough, and persistent phlegm, respectively, associated with each interquartile range (IQR) rise in O_3_ concentration. Interaction analyses showed stronger adverse effects of O_3_ on AR in children aged 7–17 years than those aged 2–6 years, while the adverse association of O_3_ with cough was more prominent in females and children aged 7–12 years than in males and children aged 2–6 and 13–17 years. This study showed that long-term exposure to ambient O_3_ was significantly associated with higher risk of AR and bronchitic symptoms in children, and the association varies across age and gender. Our findings contribute additional evidence for the importance of controlling O_3_ pollution and protecting children from O_3_ exposure.

## 1. Introduction

Ozone (O_3_) is one of the most serious air pollutants [1,2] that has adverse respiratory and cardiovascular effects [3,4]. O_3_ can reach the distal regions of human lungs due to its low water solubility and may have more adverse effects on lung function than other pollutants [5]. Previous studies suggest that the hazards of O_3_ include deep penetration, bronchial and bronchiolar injury, and tissue hypoxia [6], which are associated with higher risk of diseases such as allergic rhinitis (AR), and respiratory symptoms such as cough, wheezing, or phlegm, and decreased lung function [7]. China is suffering from severe O_3_ pollution; O_3_ concentrations in China have been continuously increasing [8]. According to a comprehensive government report monitoring 337 Chinese cities in 2019, the mean daily maximum 8-h average O_3_ concentration was 148 μg/m^3^ in China, with 30.6% of the cities showing a higher level of O_3_ than Standard II (160μg/m^3^) [9]. 

The prevalence of allergic diseases in Chinese children has increased dramatically in recent decades. For instance, allergic diseases increased by nearly five times between 1990 and 2011 [10] and the prevalence is projected to keep rising in the next 20 years, posing a higher burden of allergic diseases on the health care system [11]. Importantly, respiratory health in early life may have lifelong impacts on lung health and life expectancy [12,13]. However, evidence for the long-term effects of O_3_ on children’s respiratory conditions, especially for AR, is limited. Existing studies provide inconsistent results for the association of O_3_ and AR [14,15,16,17,18,19] and bronchitic symptoms [20,21,22,23,24,25,26]. For example, a cohort study among 1286 Canadian children [16] showed that O_3_ exposure at birth was associated with AR with its hazard ratio (HR) of 1.15 (95% CI: 1.00–1.31) for an interquartile range increase (IQR) in O_3_, whereas a study in Norway and Sweden [14] and a study from Taipei [15] suggested that O_3_ exposure at susceptibility windows (lifetime, adulthood, 0–10 years and 10–18 years) was not associated with AR. As for bronchitic symptoms, some research indicated a significant adverse effect of O_3_ on cough and phlegm [21,22,23,24,26], but studies of 1325 Chinese [20] and 3676 Southern California students [25] reported opposite results. Given these mixed results, it is important to explore further the relationship between long-term O_3_ and AR and bronchitic symptoms such as cough and phlegm in large populations such as China where severe air pollution coincides with a heavy burden of children’s respiratory disease.

Based on the large population-based study of Seven Northeastern Cities (SNEC) in China, we aim to evaluate the association of long-term exposure to ambient O_3_ with AR and bronchitic symptoms in children between 2 and 17 years of age.

## 2. Materials and Methods

### 2.1. Study Population

**Figure 1:**
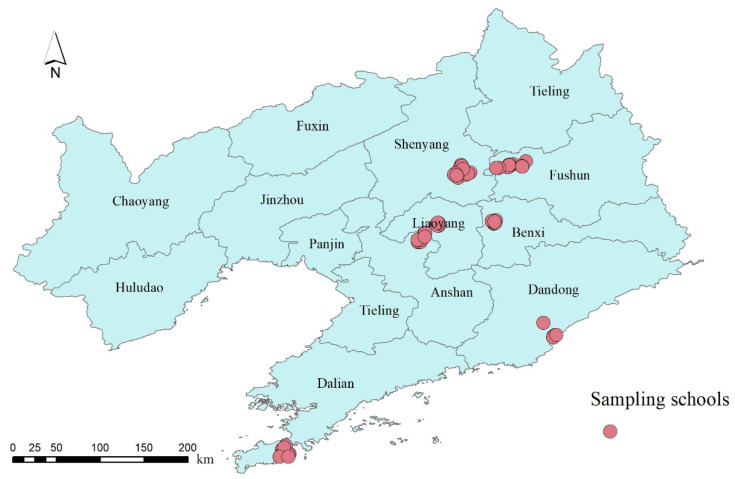
The map of Liaoning Province that shows the distribution of sampled schools in seven cities.

The SNEC study is a large cross-sectional study conducted from April 2012 to June 2013. This study aimed to examine effects of air pollution on children’s health in Liaoning Province, which is a highly industrialized region in northeastern China [27]. We first selected 27 urban districts from 7 cities (6 in Shenyang, 5 in Dalian, 4 in Fushun, 3 in Anshan, 3 in Benxi, 3 in Dandong, and 3 in Liaoyang). Then, schools were randomly selected within 1 km of each air monitoring station in each district, including 1–2 kindergartens, 1–2 elementary schools, and 1–2 middle schools, as shown in Figure 1. For each school, 1–2 classes in each grade were randomly selected and all children in each selected class were finally investigated. Informed consents and questionnaires were obtained from the children’s parents. Participants were restricted to children presently living in a residence within 1.5 miles from the monitoring station and school for at least two years. Finally, a total of 59,754 valid questionnaires out of 68,647 was received, with a response rate of 87.05%. The flow chart of participant recruitment is shown in Figure S1 in the Supplementary Material. This study obtained ethics approval from the Human Ethics Committee of Sun Yat-sen University. A written informed consent was obtained from the parent/guardian of each child before the investigation.

### 2.2. Measurement

#### 2.2.1. Outcomes

Information for respiratory conditions was collected using the Epidemiologic Standardization Project Questionnaire of the American Thoracic Society (ATS-DLD-78-A) [28]. The Chinese language version of ATS has been validated and used in numerous epidemiological studies [29,30,31]. We collected detailed information on respiratory diseases and symptoms including doctor-diagnosed AR, persistent cough, and persistent phlegm. Doctor-diagnosed AR was defined as a positive response to the question “Has your child had any episodes of allergic rhinitis within the past two years?”, with a doctor’s certificate. The measurement of persistent cough was per the responses to several cough-related questions, which indicated a cough on most days (≥4 days per week) for ≥3 months, during the past 12 months, with an infection or not. Persistent phlegm was defined per responses to several phlegm-related questions, which indicated congestion or presence of chest phlegm, sputum, or mucus on most days (≥4 days per week) for ≥3 months, with or without an infection, during the past 12 months. More details for data collection and questionnaires have been previously reported [29,32].

#### 2.2.2. Ambient O_3_ Exposure Assessment

Levels of participants’ exposure to ambient O_3_ were estimated using a satellite-based random forest approach, which has been previously reported [33]. We considered participants’ 4-year exposure to ambient O_3_ before the investigation. Therefore, we estimated individuals’ O_3_ exposure during 2008–2011 according to their residential addresses. In brief, we collected daily data on max 8-h average ozone concentrations of surface O_3_ during 2014–2019 from 1624 sites of the China National Environmental Monitoring Centre (CNEMC) (http://www.cnemc.cn/, accessed on 5 September 2020). Reanalysis data on O3 column amount were obtained from the National Aeronautics and Space Administration (NASA) website (https://disc.gsfc.nasa.gov/datasets?Project=MERRA-2, accessed on 5 September 2020). Data on other spatial and temporal predictors were also collected, including meteorological conditions (e.g., temperature, precipitation and shortwave solar radiation), vegetation index, land cover types and day of the year. We developed an iterative random forest model to estimate surface O_3_. The results of 10-fold cross-validation showed the estimated O_3_ explained 84% of ground-level measurements. Daily max 8-h average ozone product across China was estimated during 2008–2019 at a spatial resolution of 0.0625°. Levels of individual-level exposure to O_3_ were extracted from the grid data of O_3_ estimation according to their residential addresses and date of investigation.

#### 2.2.3. Covariates

A range of potential confounders was considered in this study, guided by relevant scientific literature [16,34,35]. Personal covariates included age, gender, obesity, low birth weight (birth weight < 2500 g), premature delivery (<37 weeks’ gestation), breastfeeding (more than 4 months, 1: yes, 2: no) and exercise time per week (hours). Parental covariates included parental educational attainment (defined as the highest completed education level of either parent; 0: high school or higher, 1: middle school or lower), household income (0: ≤9999 Yuan, 1: 10,000–29,999 Yuan, 2: 30,000–100,000 Yuan, 3: >100,000 Yuan) and family allergic history (0: No, 1: Yes). Environmental covariates included environmental tobacco smoke exposure (0: No, 1: Yes, defined as living with any parent who smoked at least one cigarette per day), and residence floor area per person. According to the Centers for Disease Control and Prevention (CDC) BMI growth charts criteria, obesity was defined as body mass index (BMI: weight divided by height squared (kg/m^2^)) greater than the age- and sex-specific 95th percentile [36]. A family allergic history was defined as any biological parent or grandparent having been diagnosed with allergic diseases, including AR, asthma and conjunctivitis.

### 2.3. Statistical Analysis

Considering the potential regional differences in the associations of O_3_ level with AR and other bronchitic symptoms, a mixed-effects logistic regression model was employed to estimate the association of O_3_ level with AR and other respiratory symptoms, with city as a random effect term and other variables as fixed effect terms. Crude models included levels of O_3_ and a random effect term of city. Adjusted models further included age, gender, parental education, obesity, low birth weight, premature delivery, household income, environmental tobacco smoke exposure, breastfeeding, exercise time per week, residence floor area per person and family allergic history.

Interaction analyses were additionally performed with interaction terms of age, gender, family allergic history, obesity, breastfeeding, premature delivery, low birth weight and environmental tobacco smoke exposure separately. All results were presented as odds ratios (ORs) and corresponding 95% confidence intervals (CIs) for AR and respiratory conditions associated with each IQR change in ambient O_3_ concentration. To check the robustness of our results, we tested the non-linear relationships between O_3_ and respiratory conditions, using a categorical variable of O_3_ divided by quantiles. Moreover, two-pollutant models were developed by additionally controlling for PM_2.5_ in the adjusted models. The mixed-effect logistic models were performed using lme4 package in R version 4.0.2.

## 3. Results

### 3.1. Study Population

As shown in Table 1, the mean age of all participants was 10.31 (standard deviation (SD) = 3.60) years and 50.64% of them were boys. The prevalence of AR, persistent cough and persistent phlegm was 5.33%, 6.70% and 3.12%, respectively. A higher proportion of boys was observed in children with respiratory conditions than those without (56.92% vs. 49.77%). In addition, children with respiratory conditions had less exercise time (5.96 h vs. 6.65 h per week), higher parental educational attainment, higher obesity rate and higher fractions of low birth weight and premature delivery. They were also more likely to have environmental tobacco smoke exposure and family allergic history.

### 3.2. Ambient O_3_ Exposure

A summary of estimated concentrations of O_3_ in 7 cities during 2008–2011 is shown in Table 2. The mean O_3_ level of the seven cities was 89.19 µg/m^3^ (SD = 2.27, IQR = 3.20). Dalian had the highest O_3_ concentration among 7 cities, with a mean level of 92.54 µg/m^3^ (SD = 0.21, IQR = 0.39), whereas Benxi had the lowest level of 85.33 µg/m^3^ (SD = 1.19, IQR = 0.76).

### 3.3. Associations between O_3_ and AR and Bronchitic Symptoms

**Figure 2:**
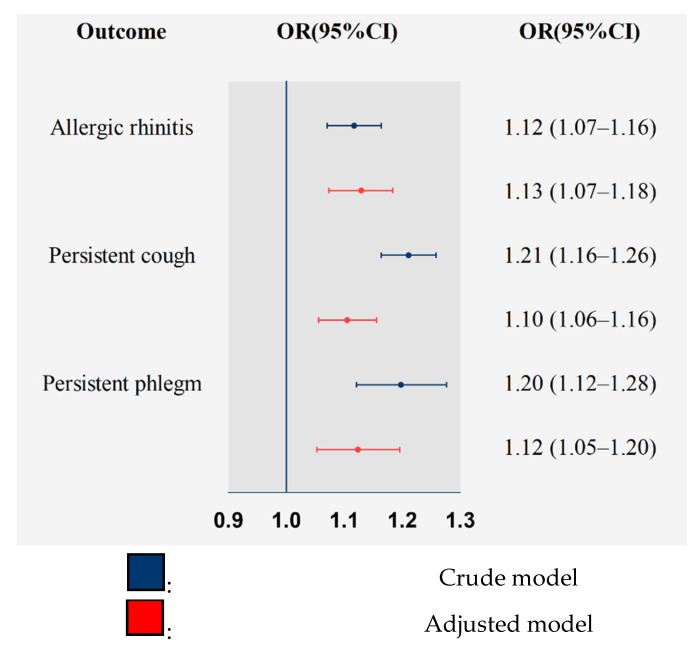
General linear mixed model regression analyses of doctor-diagnosed AR (3186 cases and 56,568 controls), Persistent phlegm (1865 cases and 57,889 controls) and Persistent cough (4004 cases and 55,750 controls) in relation to O_3_ exposure.

The results are presented in Figure 2 for the associations between O_3_ and AR and bronchitic symptoms. They indicate that long-term exposure to O_3_ was significantly associated with higher risks of respiratory conditions. In crude models, the ORs (95% CI) of AR, persistent cough and persistent phlegm associated with per IQR increase in O_3_ were 1.12 (1.07–1.16), 1.21 (1.16–1.26), and 1.20 (1.12–1.28). After controlling for potential confounders, the OR (95% CI) of AR, persistent cough, and persistent phlegm were 1.13 (1.07–1.18), 1.10 (1.06–1.16), and 1.12 (1.05–1.20), respectively.

The results of interaction analyses are shown in Table 3. Significant interaction effects of age in the association between O_3_ with AR and cough were observed. The ORs (95% CIs) of AR were 0.96 (0.85–1.08), 1.16 (1.07–1.24), and 1.14 (1.05–1.24) for individuals aged 2–6 years, 7–12 years and 13–17 years, respectively, with the *p*-value for interaction <0.05. Greater risk of cough was observed for individuals between 7–12 years (OR and 95% CI: 1.23, 1.16–1.30; *p*-value for interaction = 0.008) than other age groups, and for boys (OR and 95% CI: 1.06, 0.99–1.13; *p*-value for interaction = 0.043) than girls. No significant interaction effects were observed for other interaction terms.

The results of the sensitivity analysis are shown in Tables S1 and S2 in the Supplementary Material. Significant effects of O_3_ on AR and bronchitic symptoms were also observed in the non-linear model. The results for the associations did not substantially change by further controlling for PM_2.5_.

## 4. Discussion

Respiratory health in early life portends long-term impacts on lung health and life expectancy. Despite its importance, evidence is limited and mixed for the long-term effects of O_3_ on children’s respiratory conditions, especially for AR. In this study, we examined the associations between exposure to ambient O_3_ pollution and respiratory health of children in Northeast China. It was observed that long-term exposure to ambient O_3_ pollution was significantly associated with higher risk of AR and bronchitic symptoms, especially for cough. This association was more pronounced in children aged 7–17 years and girls. The severe O_3_ pollution and high prevalence of AR and bronchitic symptoms in northeastern China deserve more attention.

### 4.1. Associations between O_3_ and AR and Bronchitic Symptoms

The harmful effects of O_3_ on AR were previously reported. However, most evidence was for short exposure [19,37,38], and studies on long-term exposure of O_3_ are scarce. One study of 1286 Canadian children [16] suggested O_3_ concentration at birth was associated with higher risk of AR (HR and 95% CI per IQR: 1.15 (1.00–1.31)) [16]. A birth cohort study in Germany reported OR (per IQR increase in O_3_) was 1.30 (95% CI: 1.02, 1.64) [39]. However, a few studies showed results inconsistent with our study [14,15]. For example, one study of 3482 adults born after 1975 in Norway and Sweden found that O_3_ exposure was not significantly associated with AR (OR (95% CI) per 10 μg/m^3^ increase in O_3_: 0–10 years: 0.99 (0.75–1.32); 10–18 years: 1.10 (0.83–1.46); lifelong: 1.09 (0.80–1.48)) [14]. As for bronchitic symptoms, a cohort study of 4602 California children showed that reduction in O_3_ was associated with a decrease in prevalence of bronchitic symptoms with an OR (per median decreases in O_3_ based on the average changes during study period) of 0.66 (95% CI, 0.50–0.86) [22]. However, inconsistencies remain in existing studies. The results for a prospective cohort study in Taiyuan China suggested that the association between O_3_ outside the school and dry cough at night was not significant (OR and 95% CI for per 10 μg/m^3^ increase in O_3_: 0.81 (0.45–1.45)) [20]. Several reasons may explain the difference in our findings compared with previous studies, including the use of different definitions of outcomes, potential confounders considered and the exposure assessment method.

### 4.2. Potential Biological Mechanisms

The mechanisms for respiratory effects of O_3_ have not been sufficiently studied. Potential mechanisms reported by previous studies mainly focused on the Th2-dominated immune response [40]. Specifically, in rats with AR modeled in three steps of sensitization, challenge and ovalbumin [41], O_3_ showed significant impacts on the elevation of cytokine proteins such as interleukin-5 (IL-5), IL-13 and eotaxin in nasal lavage fluid, revealing a Th2-dominant immune response. For bronchitic symptoms, the main mechanisms included eosinophil-associated inflammatory responses, oxidative stress and neuroimmune pathways. Studies have found that the activation of adrenergic receptors and glucocorticoid receptors is necessary in mediating ozone-induced lung inflammation [42]. In a study of a mouse model of Aspergillus fumigatus (Af) sensitization, O_3_ exposure at 3.0 ppm for 2 h exacerbated airway reactivity by increasing eosinophil viability and inhibiting eosinophil apoptosis, accompanied by elevated levels of IL-5, granulocyte-macrophage-colony-stimulating factor (GM-CSF) and G-CSF proteins [43]. In addition, O_3_ exposure has effects on oxidative stress and numerous intracellular signaling pathways [44], probably with neuro-immune interaction [45], which may irritate and cause bronchitic symptoms such as coughing and irritated secretion causing phlegm [46].

In the gender interaction findings, girls exposed to relatively high levels of O_3_ may be at greater risk of coughing and phlegm. Experimental evidence supports the notion that girls possess a relatively higher risk when exposed to O_3_ due to sex differences in IL-6 and specific MicroRNAs (miRNA) pathways [18,47].

### 4.3. Significance for Public Health

Based on our findings, we broadly recommend government reduce O_3_ levels in cities to help prevent AR and bronchitic symptoms in children. Ground level O_3_ mainly comes from the photochemical reactions of volatile organic compounds (VOCs) and nitrogen oxides (NO_X_) [48]. To reduce levels of O_3_ pollution, measures should be taken to reduce NOx and VOC emissions, especially from industry [49] and inter-city transport [50]. For industry, sources of pollution can be reduced through process improvements such as substitution of raw materials and pre-discharge treatment. For city transport, environmentally friendly vehicles such as electric cars, buses, and light rail should be encouraged and incentivized. In addition, a stricter emission standard is needed to reduce levels of O_3_ concentrations and synergistically manage air pollutants from multiple sources (e.g., PM_2.5_ and O_3_, traffic burden and traffic pollution, multiple industrial pollutants, etc.). Individuals should avoid prolonged outdoor activities when the Air Quality Index of Ozone reaches an unhealthy level and try to schedule outdoor activities in the early morning or evening when sunlight is weak and ozone levels are low [51]. According to guidance from the United States Environmental Protection Agency (EPA), members of sensitive groups, such as people with lung disease, older adults, children and teenagers, should pay extra attention to avoid high O_3_ exposure. 

### 4.4. Advantages and Limitations

A significant strength of this study is the large sample size and good representation of participants, which covered 94 kindergartens and schools in 27 districts in northeastern China. In addition, schools have close contact with parents and hold regular parent–teacher meetings, resulting in a relatively high response rate to the survey. In addition, we used the most recent data and random forest models to estimate O_3_ concentrations in the study region, which possessed higher accuracy and spatial resolution than previous studies. 

Nevertheless, our study still has several limitations. First, as a cross-sectional study, we cannot confirm the causal relationships between O_3_ and AR and bronchitic symptoms. Furthermore, misclassification may occur, as it is possible that a participant had mild symptoms at the time but had not yet sought a medical diagnosis. In addition, participants’ exposure to O_3_ was estimated using the satellite-based random forest model based on ground-level monitoring data. However, some individual-level factors were not considered, such as living environment and daily activity, which may have an impact on the exposure assessment. In addition, residual confounding is possible due to the lack of information on environmental and individual confounders such as weather conditions and temporal activity patterns of the participants. Moreover, we were unable to distinguish between infectious and allergic bronchitic symptoms in this study, as we had no access to their detailed information on clinical diagnosis. AR in this study was obtained using questionnaires confirmed by a doctor’s certificate. We had no access to their reports of diagnostic tests, such as skin prick testing or antigen-specific immunoglobulin E testing, which may be considered in our future studies.

## 5. Conclusions

By using remote sensing data for O_3_ estimation, our study was the first in China that discovered that long-term ambient O_3_ appears to be associated with children’s AR and bronchitic symptoms’ prevalence, and which indicated children aged 7–17 years and female as relatively higher risk groups. Given its size and scope, our study provides a valuable evidence base that can draw future attention to the hazards of O_3_. Based on the findings of this study, governments and individuals should pay more attention to ambient O_3_ pollution and take effective measures to protect children from exposure to O_3_. More studies should be conducted in the future to examine relationships between long-term O_3_ exposure and AR and bronchitic symptoms in different regions and among different populations, using a prospective design and a more advanced exposure assessment method.

## Figures and Tables

**Table 1:** Characteristics of study population in the Seven Northeastern Cities (SNEC) Study.

Variables	Participants with at Least One of the Diseases Involved ^#^ (n = 7274)	Participants without Any of the Diseases Involved ^#^ (n = 52,480)	Total (n = 59,754)	*p*-Value *
Age (years), mean (SD)	10.02 (3.63)	10.35 (3.59)	10.31 (3.60)	<0.001
Gender				<0.001
Boys	4140 (56.92)	26,120 (49.77)	30,260 (50.64)	
Girls	3134 (43.08)	26,360 (50.23)	29,494 (49.36)	
Height (cm), mean (SD)	142.74 (22.29)	144.13 (21.65)	143.96 (21.73)	<0.001
Weight (kg), mean (SD)	39.85 (16.69)	40.04 (16.13)	40.02 (16.20)	0.356
Exercise time per week (hour), mean (SD)	5.96 (7.51)	6.65 (8.03)	6.56 (7.97)	<0.001
residence floor area per person (m^2^), mean (SD)	24.52 (12.77)	23.48 (12.39)	23.60 (12.44)	<0.001
Parental education				0.005
≥high school	5430 (74.65)	38,356 (73.09)	43,786 (73.28)	
<high school	1844 (25.35)	14,124 (26.91)	15,968 (26.72)	
Obesity				<0.001
No	6646 (91.37)	48,737 (92.87)	55,383 (92.69)	
Yes	628 (8.63)	3743 (7.13)	4371 (7.31)	
Low birth weight				0.019
No	6972 (95.85)	50,595 (96.41)	57,567 (96.34)	
Yes	302 (4.15)	1885 (3.59)	2187 (3.66)	
Premature delivery				<0.001
No	6756 (92.88)	49,781 (94.86)	56,537 (94.62)	
Yes	518 (7.12)	2699 (5.14)	3217 (5.38)	
Environmental tobacco smoke exposure				<0.001
No	3456 (47.51)	28,476 (54.26)	31,932 (53.44)	
Yes	3818 (52.49)	24,004 (45.74)	27,822 (46.56)	
Breastfeeding				<0.001
No	2726 (37.48)	17,272 (32.91)	19,998 (33.47)	
Yes	4548 (62.52)	35,208 (67.09)	39,756 (66.53)	
Family allergic history				<0.001
No	4802 (66.02)	43,410 (82.72)	48,212 (80.68)	
Yes	2472 (33.98)	9070 (17.28)	11,542 (19.32)	
Family income per year				<0.001
≤9999 RMB	1655 (22.75)	10,804 (20.59)	12,459 (20.85)	
10,000–29,999 RMB	2445 (33.61)	19,725 (37.59)	22,170 (37.10)	
30,000–100,000 RMB	2560 (35.19)	18,438 (35.13)	20,998 (35.14)	
>100,000 RMB	614 (8.44)	3513 (6.69)	4127 (6.91)	

Values are n (%) except where indicated. RMB, Chinese Renminbi Yuan; SD, standard deviation. ^#^ Participants with at least one of the three diseases (allergic rhinitis, persistent cough and persistent phlegm), or without three of them. * *p*-value between children with at least one condition and without three of them, which is tested by χ^2^ test for categorical variables and Student’s *t*-test for continuous variables.

**Table 2:** O_3_ concentrations for 94 schools in northeastern China, 2008–2011.

City	2008	2009	2010	2011	Average 2008–2011
Shenyang	91.05 (0.37)	90.42 (0.38)	89.55 (0.33)	89.20 (0.25)	90.07 (0.35)
Dalian	92.92 (0.40)	94.08 (0.08)	92.23 (0.50)	91.16 (0.52)	92.62 (0.39)
Fushun	87.45 (1.29)	87.24 (1.60)	86.44 (1.44)	86.59 (1.41)	86.89 (1.43)
Anshan	91.30 (0.27)	91.30 (0.24)	89.74 (0.25)	89.55 (0.33)	90.45 (0.25)
Benxi	85.99 (0.83)	86.27 (0.70)	85.72 (0.81)	85.78 (0.68)	85.94 (0.76)
Dandong	88.57 (0.11)	88.71 (0.11)	87.89 (0.08)	87.28 (0.09)	88.11 (0.10)
Liaoyang	91.13 (0.16)	91.09 (0.05)	89.90 (0.14)	89.83 (0.16)	90.48 (0.12)
Total	90.91 (3.52)	90.31 (3.30)	89.41 (2.94)	89.08 (2.76)	89.92 (3.20)

O_3_ concentrations were described with median (IQR) (μg/m^3^). IQR: Range from 25th to 75th percentile.

**Table 3:** Changes of AR, cough and phlegm associated with per IQR increment in O_3_ exposure in interaction analysis.

	Allergic Rhinitis		Cough		Phlegm	
	OR (95% CI)	*p* *	OR (95% CI)	*p* *	OR (95% CI)	*p* *
Age						
2–6	0.96 (0.85–1.08)		1.06 (0.97–1.15)		1.14 (1.01–1.27)	
7–12	1.16 (1.07–1.24)	0.012	1.23 (1.16–1.30)	0.008	1.21 (1.15–1.28)	0.423
13–17	1.14 (1.05–1.24)	0.022	1.02 (0.93–1.12)	0.584	1.03 (0.91–1.16)	0.238
Gender						
Boys	1.09 (0.95–1.26)		0.96 (0.83–1.11)		0.96 (0.80–1.15)	
Girls	1.12 (1.05–1.19)	0.641	1.06 (0.99–1.13)	0.043	1.07 (0.98–1.16)	0.066
Family allergic history						
No	1.09 (1.03–1.16)		1.11 (1.05–1.17)		1.12 (1.04–1.21)	
Yes	1.17 (1.09–1.26)	0.131	1.09 (1.004–1.19)	0.770	1.11 (0.99–1.25)	0.890
Obesity						
No	1.12 (1.07–1.18)		1.11 (1.06–1.17)		1.14 (1.06–1.22)	
Yes	1.17 (0.98–1.39)	0.662	1.02 (0.89–1.17)	0.243	0.99 (0.81–1.2)	0.183
Breastfeeding						
No	1.13 (1.04–1.22)		1.05 (0.98–1.13)		1.07 (0.97–1.19)	
Yes	1.13 (1.06–1.20)	0.995	1.14 (1.08–1.20)	0.081	1.15 (1.06–1.24)	0.275
Premature delivery						
No	1.12 (1.07–1.18)		1.11 (1.06–1.16)		1.13 (1.06–1.21)	
Yes	1.19 (1.01–1.40)	0.510	1.07 (0.92–1.25)	0.701	1.05 (0.85–1.28)	0.481
Low birth weight						
No	1.13 (1.07–1.18)		1.10 (1.05–1.15)		1.11 (1.04–1.19)	
Yes	1.18 (0.94–1.47)	0.697	1.23 (0.99–1.53)	0.308	1.30 (1.004–1.69)	0.247
Environmental tobacco smoke exposure						
No	1.15 (1.08–1.22)		1.07 (1.01–1.14)		1.09 (0.99–1.20)	
Yes	1.10 (1.03–1.18)	0.372	1.13 (1.07–1.20)	0.216	1.15 (1.06–1.25)	0.382

* All *p*-values were interaction terms, *p* < 0.05 means significant and marked bold as well as OR and 95% CI.

## Data Availability

The data are not publicly available at this time as the data also form part of an ongoing study.

## Supplementary Materials

The following are available online at https://www.mdpi.com/article/10.3390/toxics9090221/s1, Table S1: Sensitivity analysis model through classification of O_3_ estimates into four categorical variables. Based on the main model, O_3_ was changed into four categories by median and quartiles, Table S2: Sensitivity analysis model adjusted based on the original model for PM_2.5_, Figure S1: Flow chart of inclusion process of study population.
