# Supplementary material for: Relationships between Long-Term Ozone Exposure and Allergic Rhinitis and Bronchitic Symptoms in Chinese Children
Source: Toxics. 2021 Sep 14;9(9):221. doi: 10.3390/toxics9090221 (PMC8472948; doi:10.3390/toxics9090221)
Supplement: Supplementary file 1 [file toxics-09-00221-s001.zip › toxics-1352807-supplementary.pdf]

toxics MDPI

Supplementary Materials: Relationship between long-term ambient ozone exposure and allergic rhinitis and bronchitic symptoms in children.

Pei-En Zhou, Zhengmin (Min) Qian, Stephen Edward McMillin, Michael G. Vaughn, Zhong-Yue Xie, Yu-Jie Xu, Li-Zi Lin, Li-Wen Hu, Bo-Yi Yang, Xiao-Wen Zeng, Wang-Jian Zhang, Ru-Qing Liu, Gongbo Chen and Guang-Hui Dong

Sensitivity Analysis

We first recategorized O_3 into 4 categories according to median and quartiles. The results shown in Table S1 remain significant and support increasing O_3 concentrations as a risk factor for increased risk of diseases. We then included $PM_{2.5}$ in the original model and found that O_3 was still significant as shown in Table S2. Data for $PM_{2.5}$ were obtained from ground monitoring stations.

Table S1. Sensitivity analysis model through classification of O_3 estimates into four categorical variables. Based on the main model, O_3 was changed into four categories by median and quartiles.

Outcome	Category*	OR (95%CI)	p-value
Allergic rhinitis	Q1	-	-
	Q2	1.08 (0.96-1.22)	0.206
	Q3	1.24 (1.10-1.39)	<0.001
	Q4	1.26 (1.12-1.41)	<0.001
Persistent cough	Q1	-	-
	Q2	1.08 (0.96-1.21)	0.184
	Q3	1.17 (1.05-1.29)	0.003
	Q4	1.29 (1.16-1.43)	<0.001
Persistent phlegm	Q1	-	-
	Q2	1.10 (0.93-1.29)	0.255
	Q3	1.23 (1.06-1.42)	0.006
	Q4	1.37 (1.18-1.59)	<0.001

*Based on the main model, O_3 was changed into four categories by median and quartiles. Q1-Q4: quartile 1 to quartile 4, where Q1 was set as reference group.

Table S2. Sensitivity analysis model adjusted based on the original model for $PM_{2.5}$.

Outcome	OR (95%CI)		p-value	
	Without $PM_{2.5}$	With $PM_{2.5}$	Without $PM_{2.5}$	With $PM_{2.5}$
Allergic rhinitis	1.13 (1.07-1.18)	1.13 (1.07-1.20)	<0.001	<0.001
Persistent cough	1.10 (1.06-1.16)	1.05 (1.00-1.11)	<0.001	0.049
Persistent phlegm	1.12 (1.05-1.20)	1.10 (1.02-1.18)	<0.001	0.015

PM: particulate matter.

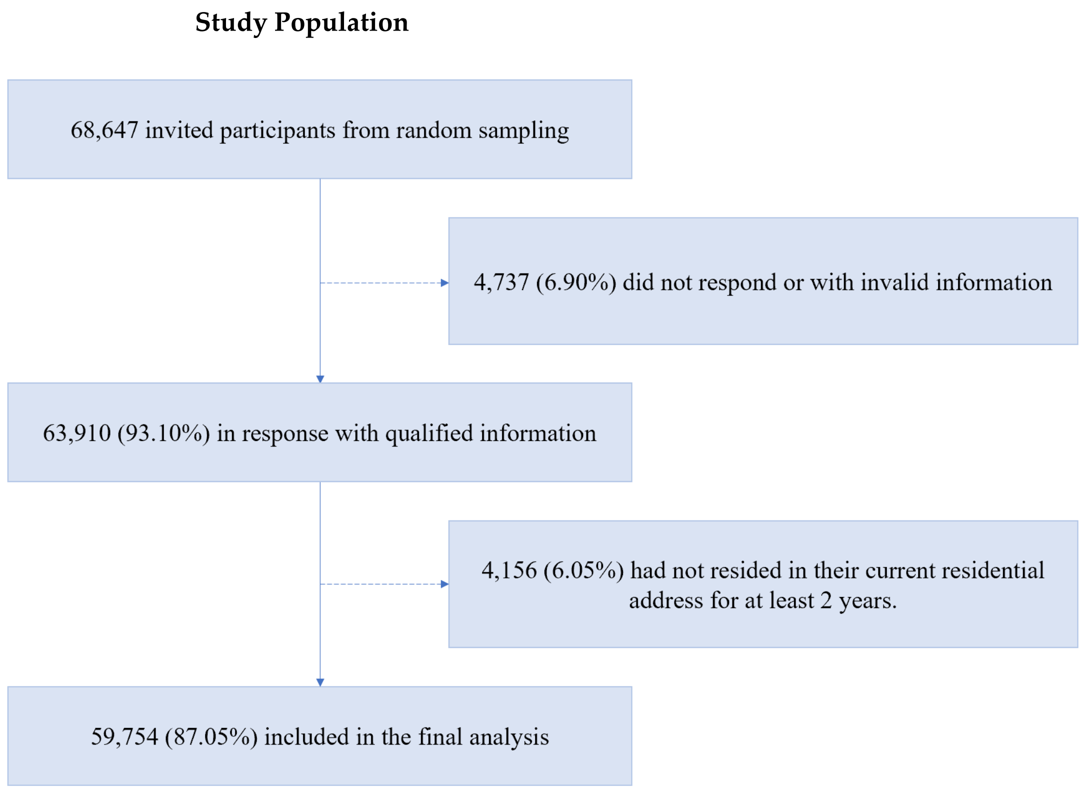

Figure S1. Flow chart of inclusion process of study population.

Questionnaire in Chinese version

辽宁省学生健康调查表

1. 学生姓名______ 2. 性别______

3. 所在学校____________年级_____班级_____

4. 出生日期______年____月____日 5. 身高：______厘米 体重：_____公斤

6. 现住址：_______市________区_________街道办事处

7. 家长联系电话____________

8. 调查表填写人与学生的亲属关系是：

1. 父亲 2. 母亲 3. 爷爷（姥爷） 4. 奶奶（姥姥） 5. 其他 9. 填表人的最高学历是：

1. 小学 2. 初中 3. 高中（中专） 4. 大学（大专）及以上 5. 其他

10. 这个孩子目前与下列哪些亲人住在一起的时间最长

1. 父亲 2. 母亲 3. 爷爷（姥爷） 4. 奶奶（姥姥） 5. 其他

11. 这个孩子所在家庭的年收入是：

1. 5000 元以下 2. 5 千-1 万 3. 1-3 万 4. 3-10 万 5. 10 万以上 9. 不知道

12. 您为这个孩子购买了下面哪种医疗保险？

1. 社会基本医疗保险 2. 商业医疗保险 3. 两者都有 4. 没有

13. 您们对这个孩子主要采用以下哪一种家庭教育方式（可以多选）？

1. 说服教育 2. 管教严厉 3. 顺其自然 4. 其它（请注明）________

14. 您的这个孩子平时认为作业负担是（幼儿不需填写）： 1. 轻松 2. 一般 3. 过重

15. 填表日期______年____月____日

下面对您的这个孩子进行有关呼吸道症状方面的调查。请您在各问题答案前的数字上打“〇”或在“___”上填写数字及文字说明，并请调查中的提示跳过某些不必回答的问题，若对某一提问不明白或因记不清楚而无法回答时，请在“否”前的数字上打“〇”。

［咳嗽］

1.1 您的这个孩子患感冒时经常咳嗽吗？ 1. 是 2. 否

1.2 即使不感冒也经常咳嗽吗？ 1. 是 2. 否

（若回答“否”，请直接回答第 2.1 项问题）

1.3 有过一年 3 个月以上，每天（一周 4 天以上）咳嗽的情况吗？ 1. 是 2. 否

1.4 多大时开始出现一年 3 个月以上，每天（一周 4 天以上）咳嗽的情况？___月或__周岁

1.5 一年 3 个月以上，每天咳嗽的情况已有几年了？ _____年 1.6 通常哪几个月咳嗽最严重？请在相应的月份上打“〇”

1 2 3 4 5 6 7 8 9 10 11 12 与月份无关

［呼哧呼哧与咳痰］

2.1 您的这个孩子患感冒时，总是呼哧呼哧的并且咳痰吗？ 1. 是 2. 否

2.2 即使不感冒，也总是呼哧呼哧的并且咳痰？ 1. 是 2. 否

（若对此问题的答案为“否”，请直接回答第 3.1 项问题）

2.3 像这种呼哧呼哧和咳痰的症状，有过一年 3 个月以上、每天（每周 4 天以上）发生的情况吗？ 1. 是 2. 否

2.4 多大时开始出现这种一年 3 个月以上、每天（每周 4 天以上）呼哧呼哧和咳痰的症状？

_____月或_____周岁

2.5 这种一年 3 个月以上、每日呼哧呼哧和咳痰的情况已经有几年了？ _____年 2.6 通常哪几个月咳痰最严重？请在相应的月份上打“〇”

1 2 3 4 5 6 7 8 9 10 11 12 与月份无关

［喘鸣］

3.1 您的这个孩子是否有过呼吸时胸中发出咝咝或呼呼的鸣笛声或口哨声（即喘鸣音）的情况？ 1. 是 2. 否

（若对此问题的答案为“否”，请直接回答第 4.1 项问题）

3.2 出现上面（问题-3.1）所说的那种喘鸣音时正在患感冒吗？ 1. 是 2. 否

3.3 不感冒时也时常出现上面（问题-3.1）所说的那种喘鸣音吗？ 1. 是 2. 否 3.4 这个孩子近两年来出现上面（问题-3.1）所说的那种喘鸣音是否超过两次？

1. 是 2. 否

3.5 这个孩子从小到现在是否有过呼吸时胸中发出咝咝或呼呼的鸣笛声或口哨声（即喘鸣音）并伴有急性呼吸困难的发作？

1. 是 2. 否

（若对此问题的答案为“否”，请直接回答第 3.7 项问题）

3.6 到现在为止，上面（问题-3.5）所说的那种情况的发作出现过 2 次以上吗？

1. 是 2. 否

3.7 这个孩子长时间玩耍或跑步后是否出现上面（问题-3.1）所说的那种喘鸣音？

1. 是 2. 否

3.8 通常哪几个月喘鸣最严重？请在相应的月份上打“○”

1 2 3 4 5 6 7 8 9 10 11 12 与月份无关

［打喷嚏、流鼻涕、鼻塞］

4.1 您的这个孩子患感冒时经常打喷嚏、流鼻涕、鼻塞吗？ 1. 是 2. 否

4.2 您的这个孩子不感冒时也经常打喷嚏、流鼻涕、鼻塞吗？ 1. 是 2. 否

［感冒及呼吸道疾病史］

5.1 近 3 年来，您的这个孩子是否有因感冒、肺病或气管不好而在家休息超过三天的情况（包括学龄前）？ 1. 是 2. 否

5.2 因上述原因（问题 5.1）这个孩子三年间平均每年在家休息几次？ _____次

5.3 近 3 年来，您的这个孩子是否有因感冒、肺病或气管不好而在家休息超过一周以上的情况（包括学龄前）？

1. 是 2. 否

（若对此问题的答案为“否”，请直接回答第 5.5 项问题）

5.4 因上述原因（问题 5.3）这个孩子三年间平均每年在家休息几次（包括学龄前）？

_____次

5.5 您的这个孩子两岁前是否因感冒而患过严重的呼吸道疾病（如气管炎、肺炎）？

1. 是 2. 否

（若对此问题的答案为“否”，请直接回答第 5.8 项问题）

5.6 请您回忆一下孩子患病时大夫诊断的病名（若得病超过一次请逐个填写）？

1.___________ 2.___________ 3.

5.7 两岁前因患严重的呼吸道疾病而住过几次医院（未住过的请填“0”）？ _____次

5.8 您的这个孩子两岁前是否有过呼吸时胸中发出咝咝或呼呼的鸣笛声或口哨声（即喘鸣音）的情况？

1. 是 2. 否

（若对此问题的答案为“否”，请直接回答第 5.10 项问题）

5.9 第一次出现喘鸣音时孩子多大？ ____月或_____周岁

5.10 您的这个孩子两岁前是否有过呼吸时胸中发出嗞嗞或呼呼的鸣笛声或口哨声（即喘鸣音）并伴有呼吸困难的情况？

1. 是　2. 否

（若对此问题的答案为“否”，请直接回答第 5.12 项问题）

5.11 第一次出现喘鸣音并伴有呼吸困难时孩子多大？ _____月或_____周岁

5.12 医生曾给您这个孩子下过哮喘（或哮喘性支气管炎或者小儿哮喘）的诊断吗？

1. 是　2. 否

（若对此问题的答案为“否”，请直接回答第 6.1 项问题）

5.13 被诊断当时，孩子呼吸是否有嗞嗞或呼呼鸣笛声或口哨声（即喘鸣音）？

1. 是　2. 否

5.14 被诊断当时，孩子呼吸是否喘鸣并伴有呼吸困难？ 1. 是　2. 否

5.15 孩子多大时由医生诊断为哮喘（或哮喘性支气管炎或者小儿哮喘）？

_____月或_____周岁

5.16 孩子的哮喘通常在哪几个月发作？请在相应的月份上打“○”

1　2　3　4　5　6　7　8　9　10　11　12　与月份无关

5.17 近两年中，孩子的哮喘发作过吗？ 1. 是　2. 否

5.18 近两年中，孩子因哮喘或喘息性支气管炎或小儿喘息而接受治疗吗？1. 是　2. 否 5.19 孩子最后一次哮喘发作时是多大年龄？ _____月或_____周岁

［其他疾病史］

6.1 医生曾诊断您这个孩子患过敏性鼻炎吗？ 1. 是　2. 否

6.2 孩子多大时由医生诊断为患过敏性鼻炎？ _____月或_____周岁

6.3 孩子的过敏性鼻炎通常在哪几个月发作？请在相应月份上打“○”

1　2　3　4　5　6　7　8　9　10　11　12　与月份无关

6.4 这两年中，孩子的过敏性鼻炎发作过吗？ 1. 是　2. 否

6.5 这两年中，孩子因过敏性鼻炎而接受过治疗吗？ 1. 是　2. 否

6.6 孩子最后一次过敏性鼻炎发作时是多大年龄？ _____月或_____周岁

6.7 医生是否检查出您这个孩子为过敏性体质？ 1. 是　2. 否

6.8 医生是否检查出您这个孩子对某种物质过敏（请逐项选择）？

1. 食物　2. 药物　3. 灰尘　4. 花粉　5. 洗涤剂　6. 其他　7. 否

6.9 请回忆一下医生是否曾诊断过您这个孩子患有下列疾病？如果有，请填上第一次患病的年龄。

病名	是否患过		首次患病的年龄
1. 麻诊	1. 是	2. 否	_____周岁
2. 百日咳	1. 是	2. 否	_____周岁
3. 支气管炎	1. 是	2. 否	_____周岁
4. 肺炎或支气管肺炎	1. 是	2. 否	_____周岁
5. 鼻窦炎	1. 是	2. 否	_____周岁
6. 湿疹	1. 是	2. 否	_____周岁
7. 过敏性结膜炎	1. 是	2. 否	_____周岁
8. 中耳炎	1. 是	2. 否	_____周岁
9. 心脏病	1. 是	2. 否	_____周岁
10. 支气管哮喘	1. 是	2. 否	_____周岁
11. 其他	1. 是	2. 否	_____周岁

6.10 近一年内，医生曾诊断您这个孩子患支气管炎吗？ 1. 是 2. 否

6.11 近一年内，医生曾诊断您这个孩子患肺炎或支气管肺炎吗？ 1. 是 2. 否

6.12 您的这个孩子曾做过扁桃体手术吗？ 1. 是 2. 否

［其他问题］

7.1 孩子出生后 3 个月内采用下列哪种哺乳方式（可以多选）？

1. 母乳 2. 牛奶或奶粉 3. 其他

7.2 若孩子是母乳喂养，那么孩子多大时断乳？ _____月或_____周岁

7.3 您的这个孩子出生时采用的是剖腹产吗？ 1. 是 2. 否

7.4 您的这个孩子是否属于早产？ 1. 是 2. 否

7.5 您的这个孩子出生时是否曾经送到过新生儿监护病房？ 1. 是 2. 否

7.6 母亲在怀孕这个孩子的时候心情如何？ 1. 好 2. 不好 7.7 母亲在怀孕这个孩子的时候年龄多大？ ______周岁

7.8 孩子的出生体重是多少？ _____斤____两

7.9 您的这个孩子是独生子或独生女吗？ 1. 是 2. 否

7.10 孩子在近三年内接种过流感疫苗吗？ 1. 是 2. 否

7.11 这个孩子平时每周在户外活动的时间是多少？ _____分钟或_____小时

［有关家庭成员的问题］

8.1 与这个孩子经常住在一起的亲属中有在家中吸烟的吗？ 1. 是 2. 否

（若对此问题的答案为“否”，请直接回答第 8.3 项问题）

8.2 他和孩子在一起的时候吸几支烟？如果他与孩子在一起时不吸烟请填上“0”支。

吸烟者与孩子的关系	和孩子在一起时吸烟支数	
	平日	节假日
父亲	___支/日	__ ____支/日
母亲	___支/日	__ ____支/日
爷爷	___支/日	__ ____支/日
奶奶	___支/日	__ ____支/日
姥爷	___支/日	__ ____支/日
姥姥	___支/日	__ ____支/日
其他（ ）	___ ___支/日	__ ____支/日

注：“其他”指伯父、叔父、姑母、舅父、姨母

8.3 母亲在怀这个孩子到出生这段时间内，家庭中或与孩子一起居住的人是否有经常在母亲面前吸烟的？

1. 是 2. 否

8.4 孩子从出生到两岁这段时间内，家庭中或与孩子一起居住的人是否有经常在孩子面前 吸烟的？

1. 是 2. 否

8.5 怀这个孩子时母亲是否吸烟？ 1. 是 2. 否

8.6 怀这个孩子时母亲是否饮酒？ 1. 是 2. 否

［家族史］

9.1 这个孩子的父亲、母亲、（外）祖父、（外）祖母、叔父（舅父）、姑母（姨母）等是否患过以下疾病。若患病的话请在表中相应的空格内画“ √ ”。

病名	父亲	母亲	爷爷	奶奶	姥爷	姥姥	其他
哮喘（支气管哮喘）							
慢性支气管炎							
肺气肿							

湿疹							
过敏性鼻炎							
过敏性结膜炎							

注：“其他”指伯父、叔父、姑母、舅父、姨母

［孩子的居住环境］

10.1 这个孩子现在的住房是哪种建筑类型？

1. 平房　2. 楼房　3. 其它

10.2 这个孩子现在的住房是哪年的建筑？ _____年

10.3 孩子的住房是在几楼？ _____楼

10.4 孩子现在居住的住房位于下列哪类区域内？

1. 居住区　2. 工业区　3. 商业区　4. 其他

10.5 孩子现在居住的住房沿街吗？ 1. 是　2. 否

10.6 孩子现在居住的住房与主要交通干线相距大约多少米？

1）＜5 米　2）5-19 米　3）20-100 米　4）＞100 米

10.7 孩子的住房与工厂的距离？

1）＜20 米　2）20-100 米　3）＞100 米　4）附近没有工厂

10.8 孩子的住房与大烟囱的距离？

1）＜20 米　2）20-100 米　3）＞100 米　4）附近没有大烟囱

10.9 孩子的住房最近 3 年内是否装修过？ 1. 是　2. 否

（若对此问题的答案为“否”，请直接回答第 10.11 项问题）

10.10 室内装修时采用了下列哪些方式（可以多选）？

1.油漆　2.地板革　3.地板　4.地毯　5.墙壁粉刷涂料或壁纸　6.购买新家具　7.其它

10.11 孩子家中有几个房间（含门厅，不含卫生间和壁橱）？ ________间

10.12 住房面积是多少平方米？ _____平方米

10.13 孩子家中共有几个人居住？ ________人

10.14 孩子家中冬天是否经常开窗换气？

1. 经常　2. 偶尔　3. 很少

10.15 孩子家中是否使用加湿器？ 1. 是　2. 否

10.16 孩子自己或与家人同住的居室的地面是什么材料（可以多选）？

1. 水泥　2. 地板　3. 地毯　4. 地砖　5. 地板革　6. 土地面

10.17 孩子现在在家中冬季里用什么方式取暖（可以多选）？

1. 暖气 2. 煤炉或火炕 3. 电暖气 4. 煤气取暖器 5. 其他

10.18 孩子家中做饭主要用哪种燃料或能源（可以多选）？

1. 煤 2. 煤气或液化气 3. 电 4. 木材 5. 煤油 6. 其他

10.19 厨房内是否安装了抽油烟机、排风扇等排气设备？ 1. 是 2. 否

10.20 做饭时，厨房内的烟对做饭者眼睛是否产生刺激症状？ 1. 是 2. 否

10.21 做饭时，家里（除了厨房以外）有多少烟？

1. 没有 2. 轻度 3. 中度 4. 重度

10.22 这个孩子家中现在是否饲养了小动物（宠物）？ 1. 是 2. 否（若对此问题的答案为“否”，请直接回答第 10.26 项问题）

10.23 这个孩子家中现在饲养的各种小动物（宠物）的数量是多少？ ________个

10.24 这个孩子家里现在饲养了下列小动物吗？

1. 猫 2. 狗 3. 鸟 4. 鸡、鸭、鹅 5. 其他 6. 未养任何小动物

10.25 这个孩子晚上睡觉时是否经常与小动物睡在一起？ 1. 是 2. 否

10.26 孩子从出生到 2 岁这段时间内家中是否饲养了小动物（宠物）？ 1. 是 2. 否

10.27 母亲在怀孕这个孩子时家中是否饲养了小动物（宠物）？ 1. 是 2. 否

10.28 这个孩子家里的墙壁上是否因屋漏雨或漏水而出现发霉或长毛？ 1. 是 2. 否（若对此问题的答案为“否”，请直接回答第 10.29 项问题）

10.29 家里的墙壁共有几处地方出现发霉或长毛？ _______处

10.30 这个孩子家里是否有蟑螂出现？ 1. 是 2. 否

10.31 这个孩子是否自己睡单独房间？ 1. 是 2. 否

10.32 这个孩子是否自己睡一床？ 1. 是 2. 否

10.33 您的这个孩子是否曾经到游泳馆游泳？ 1. 是 2. 否

10.34 您的这个孩子第一次到游泳馆游泳的年龄是多大？ _______周岁

10.35 这个孩子睡觉的房间里是否安装了电视？ 1. 是 2. 否

10.36 您的这个孩子平时每天看电视的时间大约是多少？ _____分钟或_____小时

10.37 这个孩子每天上学采用何种交通工具？

1. 步行 2. 自行车（包括成人接送） 3. 摩托车 4. 公共汽车 5. 小汽车 6. 其它

10.38 孩子从家到学校在路上需用多长时间？ _____分钟

[孩子的睡眠状况]

睡眠是影响学生身体健康的重要因素，充足的睡眠可保证儿童具有良好的学习状态。以下 26 个问题是了解您的孩子在过去 6 个月中的睡眠状况，请您从“从不”、“偶尔”、“有时”、“经常”、“总是”五个答案中选择一个最合适您孩子情况的答案。请在相应答案上打
“〇”。

1.平时您的孩子晚上睡眠一般多少时间（小时）？	>9	8-9	7-8	5-7	<5
2.平时您的孩子上床后需要多少分钟才能入睡？	<15	15-30	30-45	45-60	>60
以下问题选项注释：“从不=从来没有出现过”；“偶尔=每月出现 1-2 次”；“有时=每周出现 1-2 次”；“经常=每周出现 3-5 次”；“总是=每周出现次数>5 次”					
3. 该上床睡觉的时候，您的孩子很不情愿上床睡觉	从不	偶尔	有时	经常	总是
4. 晚上睡觉时，您的孩子比较难以入睡或入睡比较困难	从不	偶尔	有时	经常	总是
5. 孩子在入睡过程中感到焦虑或害怕	从不	偶尔	有时	经常	总是
6. 孩子在入睡过程中出现突然惊跳或抽动身体某个部位	从不	偶尔	有时	经常	总是
7. 孩子在入睡过程中总是重复某个动作如摇摆或翻身	从不	偶尔	有时	经常	总是
8. 孩子在入睡过程中头脑中出现梦境般的场景	从不	偶尔	有时	经常	总是
9. 孩子在入睡过程中身上出许多汗	从不	偶尔	有时	经常	总是
10. 每晚孩子醒来超过两次	从不	偶尔	有时	经常	总是
11. 孩子半夜睡醒后很难再次入睡	从不	偶尔	有时	经常	总是
12. 孩子在睡觉时经常翻身或踢腿把被踹掉	从不	偶尔	有时	经常	总是
13. 晚上睡觉时孩子出现呼吸困难或呼吸不畅	从不	偶尔	有时	经常	总是
14. 晚上睡觉时孩子出现张口呼吸或呼吸暂停现象	从不	偶尔	有时	经常	总是
15. 孩子睡觉时打呼噜	从不	偶尔	有时	经常	总是
16. 在晚上睡觉时，孩子身上过度出汗	从不	偶尔	有时	经常	总是
17. 您曾经看到孩子梦游	从不	偶尔	有时	经常	总是
18. 您曾经听到孩子睡觉时说梦话	从不	偶尔	有时	经常	总是
19. 孩子睡觉时磨牙	从不	偶尔	有时	经常	总是
20. 孩子突然尖叫着或表情迷茫的醒来，而你无法与他（她）交谈，但孩子早上醒来却忘记了	从不	偶尔	有时	经常	总是
21. 孩子晚上睡觉时做噩梦，但第二天醒来后却不记得	从不	偶尔	有时	经常	总是
22. 早上孩子很难醒来	从不	偶尔	有时	经常	总是
23. 早上醒来，孩子感到非常疲倦	从不	偶尔	有时	经常	总是

24. 早上孩子醒来后感到无法活动	从不	偶尔	有时	经常	总是
25. 孩子白天打瞌睡	从不	偶尔	有时	经常	总是
26. 孩子在不适合睡觉的环境中能够突然入睡	从不	偶尔	有时	经常	总是

［孩子的学习行为］

孩子的日常行为举止可影响学习态度和学习效率，以下 20 个问题是了解您的孩子平时或一贯表现情况的描述，请您根据了解的信息从“无”、“稍有”、“较多”、“很多”四个答案中选择一个最合适您孩子情况的答案。请在相应答案上打“〇”。

1. 上课时坐立不安	无	稍有	较多	很多
2. 上课时经常讲话	无	稍有	较多	很多
3. 上课时小动作多	无	稍有	较多	很多
4. 发言不举手	无	稍有	较多	很多
5. 不专心，东张西望，易因外界干扰而分心	无	稍有	较多	很多
6. 情绪变化快，容易与别人争吵	无	稍有	较多	很多
7. 常激惹别人，干扰别人活动	无	稍有	较多	很多
8. 不能平心静气玩耍	无	稍有	较多	很多
9. 做事心血来潮，想做什么就做什么，往往有始无终	无	稍有	较多	很多
10. 做事不计后果如何	无	稍有	较多	很多
11. 随便拿父母钞票，或在外偷窃	无	稍有	较多	很多
12. 丢三落四，记忆力差	无	稍有	较多	很多
13. 学习成绩差	无	稍有	较多	很多
14. 说谎、骂人打架	无	稍有	较多	很多
15. 在游戏或集体活动时不能耐心地排队等候上场	无	稍有	较多	很多
16. 常常别人问话未完即抢着回答	无	稍有	较多	很多
17. 难以按照别人的指示去做事	无	稍有	较多	很多
18. 在做作业或游戏中难以保持注意力	无	稍有	较多	很多
19. 别人和他讲话时常似听非听	无	稍有	较多	很多
20. 常常参与危险活动而不考虑后果，如乱跑到街上而不顾周围等	无	稍有	较多	很多

调查到此结束，请您从头检查一下是否有遗漏和错误。

非常感谢您认真帮助我们完成这次调查。
